# Supplementary material for: Pandemic preparedness in shaping psychosocial working conditions – insights for occupational safety and health from a longitudinal mixed-methods study during the COVID-19 pandemic at six company sites of one organization in Germany
Source: PLoS One. 2025 Aug 11;20(8):e0328410. doi: 10.1371/journal.pone.0328410 (PMC12338823; doi:10.1371/journal.pone.0328410)
Supplement: S1 Table — (PDF) [file pone.0328410.s001.pdf]

Supporting Information

Pandemic preparedness in shaping psychosocial working conditions – insights for occupational safety and health from a longitudinal mixed-methods study during the COVID-19 pandemic at six company sites of one organization in Germany

S1 Table. Items used to assess the perceived psychosocial demands from aspects relating to work organization, work environment, work content, and social relations in the workplace. [The original German wording of the items is printed in italics.]

Below, we ask you to assess your work situation at two time points: How did you experience your work situation BEFORE the COVID-19 pandemic, and how do you feel CURRENTLY? The time "BEFORE the COVID-19 pandemic" describes your usual work situation before the COVID-19 pandemic, "CURRENTLY" describes your work situation now. Please rate each of the following stress factors and resources based on these two perspectives. *Im Folgenden möchten wir Sie weiterhin bitten, Ihre Arbeitssituation zu zwei Zeitpunkten einzuschätzen: wie war es VOR der Coronavirus-Pandemie und wie erleben Sie Ihre Arbeitssituation AKTUELL? Der Zeitraum "vor-Corona" meint Ihre normale Arbeitssituation, so wie sie vor der Coronavirus-Pandemie war. Der Zeitraum "aktuell" meint Ihre jetzige Arbeitssituation, so wie Sie sie derzeit erleben. Bitte bewerten Sie die gleichen Aussagen jeweils aus diesen beiden Perspektiven. Weiter geht es nun mit verschiedenen Aspekten am Arbeitsplatz, die sowohl Belastungen als auch Unterstützungsmöglichkeiten in den Blick nehmen.*

[a] I have too much work to do. "strongly disagree" – "fully agree" (1-5). *Ich habe zu viel Arbeit. „Trifft gar nicht zu" – „Trifft völlig zu" (1-5).*<br>[a] I often feel like I am under time pressure. "strongly disagree" – "fully agree" (1-5). *Ich stehe häufig unter Zeitdruck. „Trifft gar nicht zu" – „Trifft völlig zu" (1-5).*<br>[a] I am interrupted in my work constantly (e.g. by other people, the telephone, etc.). "strongly disagree" – "fully agree" (1-5). *Ich werde bei meiner Arbeit immer wieder unterbrochen (beispielsweise durch andere Personen, das Telefon, etc.). „Trifft gar nicht zu" – „Trifft völlig zu" (1-5).*	Work organization<br>Cronbach"s alpha = 0.8
[a] There are unfavorable environmental conditions in my workplace, e.g., noise, climate, and dust. "strongly disagree" – "fully agree" (1-5). *An meinem Arbeitsplatz gibt es ungünstige Umgebungsbedingungen, wie Lärm, Klima, Staub. „Trifft gar nicht zu" – „Trifft völlig zu" (1-5).*<br>[a] The rooms and room setup are inadequate in my workplace. "strongly disagree" – "fully agree" (1-5). *An meinem Arbeitsplatz sind Räume und Raumausstattung ungenügend. „Trifft gar nicht zu" – „Trifft völlig zu" (1-5).*<br>[a] The equipment at my workplace is inadequate (e.g. missing/unsuitable tools, inefficient operation or setup of machines, inadequate software design). "strongly disagree" – "fully agree" (1-5). *An meinem Arbeitsplatz ist die Ausstattung mit Arbeitsmitteln unzureichend (z.B. fehlendes/ungeeignetes Werkzeug, ungünstige Bedienung oder Einrichtung von Maschinen, unzureichende Softwaregestaltung). „Trifft gar nicht zu" – „Trifft völlig zu" (1-5).*	Work environment<br>Cronbach's alpha = 0.7
How much can you influence what work is assigned to you? "no influence at all" – "very a lot of influence" (1-5). *Wie viel Einfluss haben Sie darauf, welche Arbeit Ihnen zugeteilt wird? "gar keinen Einfluss" – "sehr hohen Einfluss" (1-5).*<br>To what extent can you determine the order of the work steps yourself? "no influence at all" – "very much influence" (1-5). *Wenn Sie Ihre Tätigkeit insgesamt betrachten, inwieweit können Sie die Reihenfolge der Arbeitsschritte selbst bestimmen? „gar keinen Einfluss" – „sehr hohen Einfluss" (1-5).*<br>[a] I often lack the information, materials and tools I need. "strongly disagree" – "fully agree" (1-5). *Oft fehlen mir die benötigten Informationen, Materialien und Arbeitsmittel. „Trifft gar nicht zu" – „Trifft völlig zu" (1-5).*	Work content<br>Cronbach's alpha = 0.5
I can rely on my manager when dealing with difficulties at work. "strongly disagree" – "fully agree" (1-5). *Ich kann mich auf meine/n direkte/n Vorgesetzte/n verlassen, wenn es bei der Arbeit schwierig wird. „Trifft gar nicht zu" – „Trifft völlig zu" (1-5).*<br>I can rely on my colleagues, when dealing with difficulties at work. "strongly disagree" – "fully agree" (1-5). *Ich kann mich auf meine Kollegen/Kolleginnen verlassen, wenn es bei der Arbeit schwierig wird. „Trifft gar nicht zu" – „Trifft völlig zu" (1-5).*<br>I regularly receive feedback about the quality of my work. "strongly disagree" – "fully agree" (1-5). *Ich bekomme von Vorgesetzten und Kollegen/Kolleginnen ausreichend Rückmeldung über die Qualität meiner Arbeit. „Trifft gar nicht zu" – „Trifft völlig zu" (1-5).*	Social relations<br>Cronbach's alpha = 0.7

[a] Items have been reversed for score computation.
