# Supplementary material for: Pandemic preparedness in shaping psychosocial working conditions – insights for occupational safety and health from a longitudinal mixed-methods study during the COVID-19 pandemic at six company sites of one organization in Germany
Source: PLoS One. 2025 Aug 11;20(8):e0328410. doi: 10.1371/journal.pone.0328410 (PMC12338823; doi:10.1371/journal.pone.0328410)
Supplement: S2 Table — (PDF) [file pone.0328410.s002.pdf]

Supporting Information

Pandemic preparedness in shaping psychosocial working conditions – insights for occupational safety and health from a longitudinal mixed-methods study during the COVID-19 pandemic at six company sites of one organization in Germany

S2 Table. Perceived psychosocial demands during the COVID-19 pandemic: Missing analysis of main outcome. [m=mean; sd=standard deviation; n=absolute numbers for continuous variables]

	N=322					
**Timepoint**	**T0**		**T1**		**T2**	
Characteristics	*Mean (SD)*	*N*	*Mean (SD)*	*N*	*Mean (SD)*	*N*
Work organization (no missing items)	2.79 (0.86)	314	2.78 (0.89)	320	2.65 (0.89)	318
Work organization (mean-across-available-item approach)	2.77 (0.87)	320	2.78 (0.89)	321	2.66 (0.89)	321
Work environment (no missing items)	4.05 (0.87)	306	4.11 (0.87)	320	4.12 (0.86)	320
Work environment (mean-across-available-item approach)	4.04 (0.88)	320	4.11 (0.87)	321	4.12 (0.86)	321
Work content (no missing items)	3.60 (0.75)	312	3.60 (0.75)	317	3.62 (0.75)	320
Work content (mean-across-available-item approach)	3.60 (0.75)	320	3.59 (0.76)	321	3.61 (0.75)	321
Social relation (no missing items)	3.98 (0.79)	303	3.95 (0.82)	319	3.94 (0.83)	319
Social relation (mean-across-available-item approach)	3.96 (0.82)	321	3.96 (0.82)	321	3.93 (0.83)	322

5-point Likert scale; high values represent favorable perceptions of psychosocial demands and low values represent unfavorable perceptions of psychosocial demands.
