# Supplementary material for: Pandemic preparedness in shaping psychosocial working conditions – insights for occupational safety and health from a longitudinal mixed-methods study during the COVID-19 pandemic at six company sites of one organization in Germany
Source: PLoS One. 2025 Aug 11;20(8):e0328410. doi: 10.1371/journal.pone.0328410 (PMC12338823; doi:10.1371/journal.pone.0328410)
Supplement: S3 Table — (PDF) [file pone.0328410.s003.pdf]

Supporting Information

Pandemic preparedness in shaping psychosocial working conditions – insights for occupational safety and health from a longitudinal mixed-methods study during the COVID-19 pandemic at six company sites of one organization in Germany

S3 Table. Interview guide

**Topics**<br>Questions	Time-point
**Work-related burdens in the context of the COVID-19 pandemic**	
How have work-related burdens changed in the context of the COVID-19 pandemic?<br>• What work-related burdens have re-emerged in the context of the COVID-19 pandemic?<br>• What previous work-related burdens have been eliminated in the context of the COVID-19 pandemic?<br>• What changes did you (not) expect?<br>• How has sick leave changed in connection with the pandemic?	t0
**Assessing the risk of infection in the workplace**	
Where do you see the greatest risk of infection among employees in your department?<br>• How do you assess the risk of infection among employees in your department compared to your own risk of infection?<br>• What are the most bizarre things that have happened so far in relation to the risk of infection among your employees?<br>• What are the most reliable sources of up-to-date information about the COVID-19 pandemic?	t0
**Designing working conditions in the context of the COVID-19 pandemic**	
How have working conditions been designed in your department?<br>• What expertise was used?<br>• Who was (not) involved in decision-making processes?<br>• What was your role in the process?<br>• What was the biggest challenge for you as a leader in designing working conditions in the context of the pandemic?<br>• What do you think about antibody tests for SARS-CoV-2?<br>• What role do you think they could play in shaping working conditions?<br>• What actions do you think should still be taken that have not been taken yet?<br>• What actions do you think should be avoided?	t0
**Expectations and attitudes to infection and occupational safety measures**	
Which measures do you hope will succeed the most?<br>• Where do you have to be ready to adapt quickly?<br>• Where do you expect there to be a need for improvement?	t0
**Follow-up to the previous interview**	
It's been almost exactly half a year since we spoke. So I'd like to start by asking you: How did things go in your department?<br>• What has been on your mind the most as a manager over the last six months?<br>• How is the mood among your staff?<br>• What has your experience been in terms of workers in the assembly lines being used as 'cannon fodder'?<br>• What are the issues that your employees bring up to you?<br>• Looking back over the last 6 months, where did the employees in your department experience the greatest workload?<br>• And where did you experience the greatest workload?<br>• In almost all interviews in October, there was concern that it might be difficult to continue to motivate employees to remain cautious. Has this proven to be true? [Ask ad hoc according to the answer]	t1
**Measures to combat the pandemic**	
There has been quite a bit of back and forth politically in recent months in terms of the measures to combat the pandemic. How have you dealt with this in your company?<br>• What new measures have been added?<br>• What measures have been dropped?<br>• How do you now organize the rapid tests and the vaccination?<br>• Have there been any COVID cases in your team?<br>• What is your initial experience with the reintegration of those recovering from COVID?	t1
**Leadership**	
It is said again and again that the corona virus is changing leadership. How have you experienced this so far?<br>• To what extent has the quality of communication changed?<br>• What have been the most difficult conversations you have had at work recently?<br>• How much attention is actually being paid to the topic of leadership in your work at the moment?<br>• Of course, the research project is about the corona virus and so I am asking a lot of questions relating to it. But how much attention is currently being paid to this topic at your work at the moment?<br>• As a manager, do you feel well supported by your own manager?<br>• As a manager, you most certainly had a lot to do before the pandemic. What has changed as a result of the pandemic?<br>• What is it like with new employees?	t1
**Conclusion: review and outlook**	
When you look back now after one year of the pandemic, what is the main thing that has changed today compared to the beginning of the pandemic?<br>• What do you expect will happen in the next few months?<br>• What are some things that have been introduced as a result of the coronavirus and that you would like to keep after the pandemic?	t1
