# Supplementary material for: Pandemic preparedness in shaping psychosocial working conditions – insights for occupational safety and health from a longitudinal mixed-methods study during the COVID-19 pandemic at six company sites of one organization in Germany
Source: PLoS One. 2025 Aug 11;20(8):e0328410. doi: 10.1371/journal.pone.0328410 (PMC12338823; doi:10.1371/journal.pone.0328410)
Supplement: S4 Table — (PDF) [file pone.0328410.s004.pdf]

Supporting Information

Pandemic preparedness in shaping psychosocial working conditions – insights for occupational safety and health from a longitudinal mixed-methods study during the COVID-19 pandemic at six company sites of one organization in Germany

S4 Table. Coding frame for the qualitative interview data

**Topics**<br>*Subcategories*	Definition
**Interview partners' (IP) occupation and work responsibility**	
• *Responsibilities of the interview partners*	Responsibilities of the interview partners include all work content and tasks of IPs.<br>• What tasks do the IPs have?<br>• Which areas are the IPs responsible for?
• *Description of the local context*	This category contains information about the local context of their company.
• *Specifics of the respective occupation*	Specifics of the respective occupation describes both the responsibilities of employees and the profession-specific view of the pandemic/protective measures and the associated basic attitude.
**Leadership under pandemic conditions**	
• *Concept of leadership*	This category includes the leadership role/leadership style, tasks, competencies and responsibilities of the manager, as well as the way in which these tasks are fulfilled.
• *Leadership from afar, e.g., remote working*	This category includes passages in which the interview partners talk about the pandemic-related changes in leadership, especially with regard to virtual or reduced personal contact between and with employees.
• *Leadership strategies*	This category includes references to leadership strategies as well as the assessment of their effectiveness.
**Pandemic-related changes in working conditions**	
• *Key date*	The key date describes the day mentioned by the IP when "everything started".
• *Operational measures taken*	This category describes protective measures and regulations that have been introduced and/or installed in the company during the pandemic.
• *Additional measures taken by the manager*	Measures taken by managers themselves for their own area of responsibility in addition to the measures taken by the company.
• *Challenges*	This category describes aspects perceived as challenging in the context of pandemic-related changes to working condition.
• *Effects on social relationships*	This category includes passages in which the interview partners talk about (changes in) social relationships at the workplace, such as a lack of personal contact or a tense atmosphere.
• *Temporal aspects*	All temporal aspects of the pandemic-related changes in the company, which are divided into<br>• Before the COVID-19 pandemic<br>• At the beginning of the COVID-19 pandemic<br>• Current situation during the COVID-19 pandemic<br>• Outlook towards the future
• *Effects of the pandemic*	This category includes passages in which managers take a look at the changes that will (not) outlast the pandemic. These are changes that should be maintained (to a lesser extent) and changes that should be reversed.
• *Training of new employees*	This category includes text passages in which the IPs talk about hiring and training new employees during the pandemic.
**Organizational processes for adapting working conditions**	
• *Procedure and planning*	Procedure and planning describe the processes that took place prior to the introduction of measures and regulations.
• *Decision-makers and stakeholders involved*	This category includes all stakeholders who have been involved in the procedures and planning of pandemic-related changes of working conditions.
• *Sources of information*	Sources of information describe the sources used for obtaining information during the pandemic, which can be divided into internal and external sources.
• *Negotiating*	This category includes those processes that took place during the planning of measures and working conditions with the respective stakeholders involved. Specifically, this involves all aspects of information, communication and coordination with other colleagues in connection with pandemic-related changes.
• *Continuous learning in a dynamic situation*	This category describes those processes in which managers and employees gain further insights and understanding through experience in dealing with the pandemic in the company and react to it. The category also describes the individual/collective acquisition of knowledge and skills in the context of pandemic-related changes.
• *Relaxation of measures*	This category includes text passages in which the interview partners talk about planned, considered, rejected or implemented relaxation of measures.

Supporting Information

**Response to the measures**	
• *Assessment of measures by the manager*	This category describes how managers perceive the protective measures and assess their "impact" – which measures they find good, which better/worse (hierarchically), which they want to continue and where there is room for improvement.
• *(Non-)compliance with regulations*	This category summarizes exemplary situations of compliance or non-compliance with protective measures taken.
• *Acceptance of the measures by employees*	Acceptance describes how the protective measures are perceived and accepted by employees and managers. Important: Acceptance refers to the measures and employee type refers to the pandemic in general.
• *Types of employees*	Type of employee refers to the general attitude within the pandemic and not just in relation to the measures. Possible employee types can be, e.g.:<br>• Deniers / conspiracy theorists<br>• Concerned and fearful employees
**Culture of trust**	
• *Personal responsibility of employees*	This category describes the manager's confidence and hopes that employees will act independently and make correct assessments in connection with protective measures and regulations. It also includes employees' scope for action within the specified company regulations.
• *Extensive communication culture*	This category describes the communication feature of the company towards the employees - how does the company communicate with the employees in the context of the pandemic.
• *Culture of mutual awareness*	This category describes the mutual reference to regulations as well as mutual trust and standing together during the pandemic.
**Pandemic-related workloads**	
• *For managers*	This category describes pandemic-related workloads that increase or decrease for managers.
• *For employees*	This category describes pandemic-related workloads that increase or decrease for employees.
• *Eliminated loads*	This category describes workloads that have ceased in connection with the pandemic.
• *Workloads independent of the pandemic*	This category describes workloads that are experienced independently of the pandemic.
**Experiences with COVID-19**	
• *Risk of infection in the workplace*	This category describes the assessment of the risk of infection in the workplace.
• *Risk of infection in the private environment*	This category describes the assessment of the risk of infection in the private environment.
• *Experiences with COVID-19*	This category is about the reported (own) experiences with COVID-19.
• *Relevance of COVID-19*	This category includes text passages in which interview partners talk about the actual relevance of COVID-19 in their day-to-day work (e.g. in terms of work content).
**Changes in sick leave**	
• *No change*	In this category, interview partners report that there are no pandemic-related changes in sickness rates.
• *Improvement in sick leave*	In this category, interview partners report there has been an improvement in sickness rates since the pandemic.
• *Deterioration in sick leave*	In this category, interview partners report there has been a deterioration in sickness rates since the pandemic.
• *Reintegration after COVID-19*	These are passages in which the reintegration of employees after a COVID-19 infection is discussed. The category also includes passages in which it is stated that the return to work was no different than with other illnesses.
**Attitude towards antibody tests**	
• *Significance for work processes*	This category describes the possible changes in working conditions, such as the organization of cooperation, in connection with the performance of antibody tests. Negative examples are also included here: no change possible as a result of the antibody test.
• *Data protection*	This category contains quotes in which experiences with data protection is mentioned in regards to the antibody test.
• *Assessment of data quality*	In this category the interview partners assessment of the data quality of antibody tests are described, e.g. if antibody test generates reliable results.
**Vaccination in the company**	
• *Organization of vaccination*	This category includes text passages dealing with the organization and communication in the context of company vaccinations.
• *Significance for the organization of work*	This category describes the possible changes in working conditions through vaccinations. Negative examples are also included here, e.g. that no change possible through vaccinations.
• *Data protection*	This category describes experiences that are expected/made in the respective teams with the vaccinations.
• *Assessment of effectiveness*	This category contains quotes about the assessment of the effectiveness of the vaccinations.
