# Supplementary material for: Pandemic preparedness in shaping psychosocial working conditions – insights for occupational safety and health from a longitudinal mixed-methods study during the COVID-19 pandemic at six company sites of one organization in Germany
Source: PLoS One. 2025 Aug 11;20(8):e0328410. doi: 10.1371/journal.pone.0328410 (PMC12338823; doi:10.1371/journal.pone.0328410)
Supplement: S5 Table — (PDF) [file pone.0328410.s005.pdf]

Supporting Information

Pandemic preparedness in shaping psychosocial working conditions – insights for occupational safety and health from a longitudinal mixed-methods study during the COVID-19 pandemic at six company sites of one organization in Germany

S5 Table. Sensitivity analysis of outcome variables: perceived psychosocial demands during the COVID-19 pandemic compared between all participants and participants having participated at all three timepoints (panel data). [m=mean; sd=standard deviation; n=absolute numbers for continuous variables]

**Timepoint**	**T0**		**T1**		**T2**	
Characteristics	*Mean (SD)*	*N*	*Mean (SD)*	*N*	*Mean (SD)*	*N*
**Perceived psychosocial demands during the COVID-19 pandemic**						
Work organization	2.8 (0.87)	320	2.8 (0.89)	321	2.7 (0.89)	321
	2.7 (0.92)	2410	2.7 (0.90)	2503	2.6 (0.92)	2054
Work environment	4.0 (0.88)	320	4.1 (0.87)	321	4.1 (0.86)	321
	3.9 (0.90)	2405	4.0 (0.93)	2504	4.0 (0.92)	2053
Work content	3.6 (0.75)	320	3.6 (0.76)	321	3.6 (0.75)	321
	3.5 (0.80)	2412	3.5 (0.78)	2506	3.5 (0.80)	2051
Social relation	4.0 (0.82)	321	4.0 (0.82)	321	3.9 (0.83)	322
	3.8 (0.87)	2406	3.9 (0.85)	2507	3.8 (0.86)	2055
**Perceived psychosocial demands before the COVID-19 pandemic**						
Work organization	2.7 (0.81)	320	2.7 (0.80)	321	2.6 (0.80)	320
	2.6 (0.86)	2410	2.6 (0.84)	2503	2.6 (0.85)	2054
Work environment	4.1 (0.82)	320	4.2 (0.83)	321	4.1 (0.89)	321
	4.0 (0.89)	2405	4.0 (0.03)	2504	4.0 (0.94)	2053
Work content	3.7 (0.73)	320	3.7 (0.70)	321	3.7 (0.74)	319
	3.6 (0.78)	2412	3.6 (0.75)	2506	3.6 (0.78)	2051
Social relation	4.0 (0.78)	321	4.0 (0.77)	321	4.0 (0.76)	321
	3.9 (0.83)	2406	4.0 (0.79)	2507	3.9 (0.80)	2055

5-point Likert scale; high values represent favorable perceptions of psychosocial demands and low values represent unfavorable perceptions of psychosocial demands
