# Supplementary material for: Pandemic preparedness in shaping psychosocial working conditions – insights for occupational safety and health from a longitudinal mixed-methods study during the COVID-19 pandemic at six company sites of one organization in Germany
Source: PLoS One. 2025 Aug 11;20(8):e0328410. doi: 10.1371/journal.pone.0328410 (PMC12338823; doi:10.1371/journal.pone.0328410)
Supplement: S6 Table — (PDF) [file pone.0328410.s006.pdf]

Supporting Information

Pandemic preparedness in shaping psychosocial working conditions – insights for occupational safety and health from a longitudinal mixed-methods study during the COVID-19 pandemic at six company sites of one organization in Germany

S6 Table. Quotes from qualitative interviews.

**Category** *Subtheme*	*Quote*	*Time point*
**Pandemic-related changes in working conditions**		
For employees	When looking back on the year now, it's clear that the fear at the beginning of the pandemic was much greater than it is now. In the beginning, of course, the problem was that nobody knew what was going on from a medical point of view: what's happening, how serious are the consequences, what would happen if I got infected? Then, there were the issues relating to health and physical fitness. And the second thing was, of course, jobs .... well, then the economy collapsed. There really had been predictions that the economy would collapse and we would lose an enormous number of jobs. **IP8a**<br>*Wenn ich das Jahr jetzt noch mal Revue passieren lasse, ist es natürlich schon so, dass am Anfang der Pandemie die Angst noch weitaus größer war wie aktuell. Also am Anfang war natürlich das Thema, keiner hat sich ausgekannt aus medizinischer Sicht: Was passiert, wie schwer sind die Auswirkungen, was passiert, wenn ich es bekomme? Also da waren einmal die gesundheitlichen und körperlichen Themen. Und das Zweite, was halt noch war, dass natürlich die Arbeitsplätze ... Also es ist ja dann die Wirtschaft eingebrochen. Es gab ja dann wirklich Vorhersagen, dass die Wirtschaft dann auch einbricht und wir massiv Arbeitsplätze verlieren.*	t0
	There are a lot of misunderstandings, you speak less, write more, get things wrong, and it builds up because there are still no discussions. These are the problems, and on the other hand, of course, there are similar cases that arise because there are fewer meetings when working remotely or less contact with colleagues when working at the company. Nothing is being coordinated, so there is a duplication of work, two or three people are working on the same thing and only realize it two days later. That's frustrating when you already have too much to do. **IP3a**<br>*Da geht es ganz viel um Missverständnisse, also man redet weniger, man schreibt mehr, man kriegt es in den falschen Hals, es schaukelt sich hoch, weil wieder kein Gespräch. Das sind so die Themen, und auf der anderen Seite passiert natürlich auch ganz häufig so Geschichten durch weniger Absprache im Homeoffice oder auch durch weniger Kollegenkontakt im Betrieb, gibt keine Abstimmung, daher gibt es Doppelarbeit, zwei, drei Menschen sind am Gleichen dran und bemerken das erst zwei Tage später. Das frustriert dann, wenn man eh schon zu viel hat.*	t1
	[…] There is also a psychological burden, employees report this as well. I'm exaggerating now, but I think you might understand that, they suffer from the fact that they have no direct contact with their colleagues. [...] On the flipside, however, many colleagues say: working from home is really good, like, I don't even know whether I want to go back. **IP5a**<br>*[…] Es gibt auch ein[e] psychische Belastung, also auch das berichten Mitarbeiter, dass sie sagen, ich übertreibe jetzt mal, aber Sie verstehen mich glaube ich, die darunter leiden keinen unmittelbaren Kontakt zu ihren Kollegen zu haben. […] die andere Stimme ist aber auch, eine ganze Reihe von Kollegen sagen: Homeoffice ist richtig gut, so ungefähr, ich weiß gar nicht ob ich wieder zurückkommen möchte.*	t1
For managers	For managers, I feel that their workload has increased because there is more to organize than just the day-to-day work. Things to organize like who is allowed to be on-site, which rules are to be implemented, and which additional measures need to be managed and monitored. **IP4a**<br>*Also bei Führungskräften hat sich die Belastung gefühlt erhöht, weil es mehr zu organisieren gibt über das tägliche inhaltliche Arbeiten hinaus. In Dinge organisieren, wie welche Mitarbeiter dürfen anwesend sein, welche Regeln sind umzusetzen, welche zusätzlichen Maßnahmen sind zu steuern und zu kontrollieren.*	t0
	[…] Of course, I had individual discussions with those who are not so sensitive and I said: you have to show consideration to colleagues who are more fearful, and you have to take their concerns seriously and of course try to keep your distance and wear face-masks. **IP8c**<br>*[…] Natürlich die, wo jetzt nicht so sensibel sind, sage ich mal, mit denen habe ich Einzelgespräche gemacht und habe gesagt: Ihr müsst auf die Kollegen, die jetzt eben mehr Angst haben, eingehen und deren Sorge müsst ihr ernst nehmen und versuchen, natürlich Abstand einzuhalten, Mundschutz aufzusetzen.*	t0

Supporting Information

Organizational processes to adapt working conditions		
Decision-makers and stakeholders	And, of course, it was very important to me to involve the employees on-site, as a team, in a workshop. I was there as the foreman, one of my shift supervisors was involved as well as five employees from the assembly lines and the [process] engineer. So, we designed it together. It was also important to me to involve the employees, especially in this situation, so that they can really voice their worries or fears and concerns. **IP8b**<br>*Und, das war mir sehr wichtig, ich habe natürlich die Mitarbeiter vor Ort mitgenommen, so ein Team, ein Workshop. Ich war mit dabei als Meister, dann ein Schichtführer von mir war mit dabei und fünf Mitarbeiter von den Linien und eben noch der [Prozess-]Ingenieur. Und das haben wir dann gemeinsam ja so entworfen. Es ist mir auch wichtig, gerade in der Situation die Mitarbeiter mit einzubinden, dass sie wirklich ihre Sorgen oder ihre Ängste und ihr Anliegen praktisch mit einbringen können.*	t0
	[…] So, the crisis management team basically includes various experts – there is corporate communications, [information technology] IT, [human resources] HR, the Health Safety & Environment department and the occupational safety specialists, and then whomever else might be needed. We also had labor lawyers, as well as business lawyers, because there are questions that, of course relate to a completely different – a legal – dimension, as well as the [company medical personnel] and the representatives of the [company medical personnel] team. **IP6a**<br>*[…] also der Krisenstab hat im Wesentlichen verschiedene Situationen in sich, da ist die Unternehmenskommunikation, die IT, HR, der Bereich Health Safety Environment oder auch die Arbeitssicherheitsexperten, und dann nimmt man hinzu, wen man noch teilweise braucht, wir haben auch noch Arbeitsrechtler dazu genommen, auch dann noch Wirtschaftsrechtler, weil es auch Fragen gibt, die natürlich noch eine ganz andere, eine Rechtsdimension berührt hat, und die [Betriebsärzte] und die Vertreter des [Betriebsärzte]Teams.*	t0
Procedures and planning	Firstly, I think there is no question that we have home office workplaces on a scale that we have never had before. We are familiar with this model. We had already introduced it, but of course not on this broad of a scale. **IP5c**<br>*Da ist einmal, ich glaube das ist ein No Brainer, dass wir Homeoffice-Arbeitsplätze haben in einem Umfang, wie wir das nie hatten. Das Modell kennen wir. Das hatten wir auch eingeführt, aber natürlich nicht in der Breite.*	t0
	[…] Whenever the [new COVID-19] regulation [by the government] comes into force at short notice, like, now a rapid lateral flow test would be nice, and then all of a sudden, rapid tests are mandatory three days later, and then we try to organize that. Of course, we are part of the corporate group, which is very helpful, especially when something has to be procured on a large scale. **IP5b**<br>*[…] Wenn die Regelung dann halt kurzfristig kommt, jetzt Selbsttest wäre schön, und dann heißt es drei Tage später: Und jetzt sind Selbsttests verpflichtend, und dann versuchen, das halt zu organisieren. Da hängen wir natürlich auch im [Unternehmen]-Verbund drin, was sehr hilfreich ist, gerade, wenn dann irgendwie im großen Stil irgendetwas beschafft werden muss.*	t1
	As far as protection is concerned, I think we are actually very, very well positioned here. [...] So, as I said, our [company medical personnel] is always very, very committed and makes sure that we really take advantage of all opportunities. [...] And if we have any questions, like at the beginning when people called me, all the unresolved cases, saying: Yes, I also had contact with him, with the person who tested positively, or I also have slight symptoms now, what should I do? Well, I could always call the [company medical personnel] and talk about it: What do we do now? What do we do in this case? And I definitely got a lot of support there. I thought that was really great, yes, that they were always there for me to help me with any problem. **IP7a**<br>*Was zum Schutz angeht finde ich, dass wir eigentlich sehr, sehr gut aufgestellt sind bei uns da. […] Also, wie gesagt, unsere [Betriebsärzte], ist da immer ganz, ganz engagiert und schaut, dass man da wirklich alle Möglichkeiten ausschöpft. […] Und wenn wir mal Fragen haben, also, die Leute, bei mir am Anfang, die ganzen ungeklärten Fälle, die dann angerufen haben: Ja, ich habe auch mit ihm Kontakt gehabt mit meiner positiven Person oder ich habe jetzt auch leichte Anzeichen, was soll ich machen? Also ich konnte halt jederzeit die [Betriebsärzte] anrufen, und darüber sprechen: Wie gehen wir jetzt da vor? Was machen wir jetzt in dem Fall? Und da habe ich große Unterstützung auf jeden Fall. Das fand ich echt klasse, ja, also dass die jederzeit immer erreichbar waren, um mir eigentlich bei dem Problem zu helfen.*	t1
**Culture of trust**		
Self-responsibility	And I explained many rules and hoped that the employees would take responsibility for themselves and this was confirmed, that they were able to manage themselves and be flexible. **IP4b**<br>*[…] Fand ich auch beeindruckend, dass viele Menschen sich in solchen Situationen dann komplett aktivieren und auch dazu bereit sind, für die Gesamtorganisation und für die Menschen, die dort arbeiten, wirklich, wie man das so schön formuliert, die Extrameile gehen und sich da auch anders einbringen, als man das vermeintlich erwarten würde.*	t0
Communication culture	[…] And we also started very early with communication in general, with the protective measures. This included personal letters from our management to employees, publications in our media, such as the [company newspaper], in which we regularly publish articles. And there is a weekly newsletter, one for each country and one for [the corporate group] worldwide, which we distributed. Thus, we try to share with as many employees as possible what we are working on as well as changes, including scientific findings, legal changes and so on. **IP2a**<br>*[…] Und wir haben auch sehr früh mit der Kommunikation allgemein angefangen, mit den Schutzmaßnahmen. Das ging dann über persönliche Briefe von unserer Geschäftsführung an die Mitarbeiter, über Veröffentlichungen in unseren Medien, die wir haben, wie das [Firmenblatt], wo wir regelmäßig Beiträge berichten. Und es gibt einen wöchentlichen Newsletter, einen pro Land und einen für [den Konzern] weltweit, der verschickt wird von uns. Und so versuchen wir eben, dass wir möglichst viele an dem, was wir gerade arbeiten, teilhaben lassen dann und auch an Veränderungen, auch an wissenschaftlichen Erkenntnissen, aus rechtlichen Veränderungen und so.*	t0
	However, I also believe that it is good for communication if you say quite honestly that you don't know everything and that it is therefore possible that tomorrow you will take back or correct something that you thought was good today, so that people understand that not everything is absolute and that it changes. **IP4c**<br>*Ich glaube aber, dass die Kommunikation da auch vorteilhaft ist, wenn man sehr ehrlich damit umgeht, dass man nicht alles weiß und deswegen auch es sein kann, dass man morgen etwas widerruft, was heute für gut befunden wurde oder dass man das korrigiert, dass die Leute auch verstehen, es ist nicht alles absolut und das entwickelt sich.*	t0

The interview partners covered the following perspectives: factory security service, company medical service, works council, technical operation manager, corporate financial management, human resources department, and assembly line/ manufacturing.
The original German wording of the quotes is printed in italics.
Since we focus on the overall content, we expect no loss of meaning from the authors' translation from German into English, which was done after the analysis and checked by a native speaker.
