# Supplementary material for: Pandemic preparedness in shaping psychosocial working conditions – insights for occupational safety and health from a longitudinal mixed-methods study during the COVID-19 pandemic at six company sites of one organization in Germany
Source: PLoS One. 2025 Aug 11;20(8):e0328410. doi: 10.1371/journal.pone.0328410 (PMC12338823; doi:10.1371/journal.pone.0328410)
Supplement: Attachment — Submitted filename: Response to Reviewers.pdf [file pone.0328410.s009.pdf]

Manuscript ID: PONE-D-25-00343

Title: Pandemic preparedness in shaping psychosocial working conditions – insights for occupational safety and health from a longitudinal mixed-methods study during the COVID-19 pandemic

Dear María Andrée López Gómez,

many thanks for the valuable review process and for the opportunity to submit our revised manuscript.

Please find below our answers to the reviewers' comments on our manuscript now titled '*Pandemic preparedness in shaping psychosocial working conditions – insights for occupational safety and health from a longitudinal mixed-methods study during the COVID-19 pandemic at six company sites of one organization in Germany'.*

In addition, we uploaded a marked-up copy of our manuscript to highlight the changes made, labeled 'Revised Manuscript with Track Changes' as well as an unmarked version of the revised manuscript labeled 'Manuscript'.

We checked that our manuscript meets PLOS ONE's style requirements as well as our reference list. As we identified further relevant literature which was published since we submitted this manuscript, we added one source to the reference list.

We believe that we have replied to all of the reviewers' valuable comments and suggestions. I hope to have now clarified in the abstract and the methods section where we conducted our research and that employees and managers were employed at six German company sites in the federal states of Bavaria, Baden-Wurttemberg, and Lower Saxony of one larger company group. Please see pages 2-5 for our reponses to Reviewer 1 and pages 6-28 for our responses to Reviewer 2. Thank you very much for your kind consideration and we look forward to hearing from you.

Kind regards on behalf of all authors,

Jana Soeder (Corresponding author)

Research Associate and PhD Candidate Experimental Medicine

Institute of Occupational and Social Medicine and Health Services Research

University Hospital Tübingen

Wilhelmstrasse 27

72074 Tuebingen, Germany

Dear Reviewer 1,

thank you for your time and effort in evaluating our manuscript. We have revised the manuscript based on your comments with tracked changes to highlight the revisions. The lines indicated refer to the new lines in the revised manuscript. Please find below a point-by-point response to your comments and suggestions.

Comment 1

Line 69-70: Explanation for psychosocial related work risks needed

Answer 1

Many thanks for your recommendation. We have revised the related paragraph in the introduction to provide a clearer understanding.

Changes in the manuscript

Introduction: […] In Germany, OSH is regulated by the Safety and Health at Work Act (ArbSchG) [6]. Here, a risk assessment is the first step in deriving and implementing OSH-measures aimed at preventing work-related accidents and health hazards. Possible work-related risks can be elicited by both, physical and psychosocial demands. Psychosocial demands can – depending on the individual’s preconditions – lead to either favorable (e.g., activating state) or unfavorable strain (e.g., fatigue or problems concentrating) and thus impact the individual’s health [7]. Psychosocial demands in the workplace refer to key aspects of work organization, work environment, work content, social relations, and new forms of work. An aspect of work organization, for example, is the constant interruption during work, while an aspect of work content is the degree of influence a worker has over assigned tasks. These aspects are to be considered when conducting a psychosocial risk assessment in the workplace, according to the Joint German OSH-Strategy (GDA) [7]. […] (lines 70 ff.)

Comment 2

Line 289-291: Concerns around small sample size; power analysis/controlled variability/effect size should be shared to address inclusion of small Ns

The paper either needs more justification for the inclusion/choice of various working fields or to focus on a specific field. Unclear from the paper how the different fields would experience comparable psychosocial demands, especially when some quotes reflect unique work environments. Small Ns in statistical analysis beg the question as to why you included the various fields rather than focusing on office workers for the paper. That these are a part of one organization should be explained earlier on--with a description of the company.

Additionally, more clarity on German supports for workers during the time could be helpful and should be addressed in statistical analysis. If supports vary by working field, this should also be addressed.

Answer 2

Many thanks for that comment. We can see your point and agree. We now added the effect size not only for statistically significant results but also for non-significant results, see lines 371-377 and lines 403-414. However, we would like to highlight that until now a lack of longitudinal panel studies investigating psychosocial demands during the COVID-19 pandemic exists. Especially the working field of non-healthcare organizations is under-explored as well as the differentiation between employees and managers of different occupational working fields.

We added a paragraph describing the company group, see lines 160-170. In addition, we included a paragraph to justify the inclusion of various working fields, see lines 170-178. Previous findings from the European Working Conditions Survey revealed differences between working fields with respect to adjusted working conditions and the challenges associated with them (doi: 10.2806/056613). We here went into more detail for description in lines 112 ff. We recruited from six different company sites of one larger company group which allow to differentiate and adhere to pseudonymization guidelines, even though the sample size in some working fields is small. However, it is important to consider that office and assembly line working fields typically represent the largest employee groups within a company, whereas fields such as company medical services are naturally smaller by comparison. As previous literature by Wagner et al. (doi: 10.3390/vaccines11061082) showed that occupational health personnel suffered from increased workload, especially during the roll-out phase of the COVID-19 workplace vaccination program in Germany, it seemed of utmost importance for us to include workers related to this working field in our sample if possible.

We hope to have now made clear within the title, abstract and methods section, that all employees were recruited from six different company sites of one larger company group located in Germany.

Regarding the sample size, in the overall survey, N=5,554 employees participated. For 322 out of them, longitudinal panel data could be ensured. See section ‘Results sample

characteristics' for further details, lines 274 ff.. We added an additional sensitivity analysis to show that the outcome variables did not differ substantially between participants who took part three times compared to the overall sample, see S5 Tab in the Supplementary Material. Regarding the qualitative interviews, we considered a sample of nine interview participants sufficient to reach data saturation.

Regarding the support of both the German state and the employer, we on the one hand performed a comprehensive document analysis of prevailing national and federal laws and OSH-regulations for infection control in Germany. On the other hand, the interviews helped to address how workers experienced support from both the German state (e.g., financial aid in the form of paid temporary short-time work) and their organization (e.g., adjusted work arrangements) during the pandemic. Both parts provided the contextual background for interpreting and discussing our findings. We now tried to make this more clear when describing the study design, see lines 141 ff.. In addition, we went more into detail in the results section, especially when describing the main findings of the document analysis, see lines 300 ff. Less than 7% of the participants included reported working short-time, which limited our ability to analyze differences across working fields on that topic. However, we did provide an in-depth description how infection control measures were implemented across different working fields. These findings were also more relevant to the aim of our study.

Changes in the manuscript

Results section: The median psychosocial demands from aspects of the work environment were perceived as rather favorable and stable (X2(2)=5.47; p=0.06; r=0.09), as were the perceived psychosocial demands in terms of work content (X2(2)=0.45; p=0.08; r<0.001) and social relations (X2(2)=1.70; p=0.43; r=0.003). (lines 374-377)

Results section: Overall, psychosocial demands from aspects of work environment were perceived favorably retrospectively and did not significantly differ between timepoints (X2(2)=12.2; p=0.002; r=0.02; non-significant post-hoc tests), as were psychosocial demand ratings from aspects relating to work content (X2(2)=1.11; p=0.58; r=0.002) and social relations (X2(2)=3.88; p=0.14; r=0.006). (lines 410-414)

Methods section: The longitudinal data comes from employees and managers from various working fields from a worldwide leading global supplier of technology and services. The company group can be characterized as financially well-established and having a lot of human resources. Regarding the work environment, digitalized solutions and proven communication channels were already used before the COVID-19 pandemic. All employees and managers included were employed at six German company sites in the federal states of Bavaria, Baden-Wurttemberg, and Lower Saxony. Employees for example worked at the assembly line/production, in the office, company medical service, or factory security service of the same company group. We aim to differentiate between those working fields as they show large differences in how working conditions were adapted to ensure as-safe-as-possible workplaces. While office workers were mainly affected by remote work and online meetings where possible, assembly line/manufacturing workers experienced redesigned working conditions to allow for keeping a minimum of 1.5m distance between work stations, frequent cleaning of work equipment, and decoupled break times. Company medical service personnel

in comparison were responsible for implementing SARS-CoV-2-infection control measures as well as ensuring testing and vaccination possibilities. (l. 160 ff.)

Title: Pandemic preparedness in shaping psychosocial working conditions – insights for occupational safety and health from a longitudinal mixed-methods study during the COVID-19 pandemic at six company sites of one organization in Germany (l. 1 ff.)

Results section: The here presented survey results are based on a subsample of participants (n=322 out of N=5,554) for whom all three survey observations were available. A total of 5,554 employees participated in the online survey at one, two, or all three timepoints. For 322 out of them, longitudinal panel data could be ensured. This analysis focuses on panel data, as we were primarily interested in within-person changes in psychosocial demands.

Methods section: For contextualization, we performed a comprehensive document analysis of prevailing national and federal laws and OSH-regulations for infection control in Germany. In a parallel approach, quantitative (employee survey) and qualitative data (interviews with managers) were gathered and analyzed independently. This allowed us to gain detailed insights into the company-level context in which employees and managers work. Findings from the interviews further helped to address how workers of different working fields experienced support from both the German state (e.g., financial aid in the form of paid temporary short-time work) and their organization (e.g., adjusted work arrangements) during the pandemic. (lines 138 ff.)

Results section: Fig 1 provides an overview of the main findings of the document analysis together with the data collection phases [22-24]. For example. the German social security concept included statutory employment-protection systems such as continued payment of wages in the case of temporary short-time work due to economic difficulties already before the COVID-19 pandemic [25, 26]. In response to the pandemic, the requirements for companies in Germany to receive compensation for short-time work were eased starting in March 2020. Another relevant regulation by the German Ministry for Health is that, beginning in June 2021, occupational physicians were authorized to vaccinate in the workplace as part of the national vaccination campaign [27]. (lines 300-308)

Discussion: Our quantitative data showed that 99.7% of all participants hold permanent work contracts. Thus, we were unable to compare their perceived psychosocial demands with employees holding fixed-term contracts. We can only hypothesize that the job security provided by the permanent work contracts across all working fields in combination with the possibility to work a reduced number of hours with compensation for missing net wage contributed to the stability of perceived work-related psychosocial demands. (l. 473 ff.)

Methods: While office workers were mainly affected by remote work and online meetings where possible, assembly line/manufacturing workers experienced redesigned working conditions to allow for keeping a minimum of 1.5m distance between work stations, frequent cleaning of work equipment, and decoupled break times. Company medical service personnel in comparison were responsible for implementing SARS-CoV-2-infection control measures as well as ensuring testing and vaccination possibilities. (lines 172 ff.)

Dear Reviewer 2,

thank you very much for your valuable and comprehensive feedback on our manuscript which we appreciate a lot. We have reworked the manuscript and hope that the adaptations will meet your criticism. We revised the manuscript with tracked changes to highlight the revisions. The lines indicated refer to the new lines in the revised manuscript. Please find below a point-by-point response to your comments and suggestions.

Comment 1

Introduction - Strengths:

Contextual Relevance: The inclusion of current global drivers like digitalization, ecological transformation, and demographic changes is highly relevant. The connection to how these factors impacts work environments and psychosocial risks is timely and significant.

Clear Problem Statement: The problem you aim to address is clearly articulated: the relationship between organizational responses to the pandemic and employees' perceived psychosocial demands. This is an important and under-explored area in occupational health and safety research.

Comprehensive Literature Review: You effectively refer to prior research, including the work of the ILO, OECD, and previous crises like the Ebola epidemic and the 2008 economic recession, which supports the urgency of understanding psychosocial risks in the workplace. The inclusion of both physical and psychosocial demands aligns well with the focus of your paper.

Use of Case Examples: Your examples, such as the changes in working conditions during the COVID-19 pandemic (e.g., remote work, social distancing), help to ground the theoretical framework in real-world changes. This provides practical relevance and context to your research.

Methodological Approach: The combination of qualitative and quantitative methods adds depth to your investigation. It seems like you're trying to bridge a knowledge gap by exploring both employee and manager perceptions during the pandemic.

Suggestions for Improvement:

Clarity and Flow: The first few sentences could benefit from more clarity. For example, the initial reference to ILO and OECD could be better connected to the rest of the paragraph to avoid abruptness.

"According to the International Labor Organization (ILO) and the Organization for Economic Co-operation and Development (OECD), increasing digitalization, ecological transformation, and advancing demographic change are key drivers of accelerated technological development and the constantly changing world of work."

This sentence could be shortened and made more direct for easier readability, such as:

"The International Labor Organization (ILO) and the Organization for Economic Co-operation and Development (OECD) highlight that digitalization, ecological transformation, and demographic changes are reshaping the world of work."

Answer 1

Thank you for highlighting the importance of the research topic we addressed in our study as well as our methodological approach. Thank you for this helpful remark regarding the readability. We revised the first sentence of the introduction to ensure a better understanding and easier readability.

Changes in the manuscript

Introduction: The International Labor Organization (ILO) and Organization for Economic Co-operation and Development (OECD) highlight that digitalization, ecological transformation, and advancing demographic change are reshaping the world of work […] (line 52 ff.).

Comment 2

Language and Grammar: The phrasing in certain areas could be streamlined. For example:

"Psychosocial risk factors in the workplace such as long working hours or employment uncertainties have been shown to be strongly associated with the development of health issues and chronic diseases, e.g., burnout, coronary heart diseases, and anxiety."

This could be rephrased as: "Psychosocial risk factors, including long working hours and employment uncertainty, are strongly associated with health issues such as burnout, coronary heart disease, and anxiety."

Answer 2

We revised the mentioned sentence. In addition, we went through the whole manuscript to improve its readability by shortening and rephrasing some sentences. Furthermore, a native speaker reviewed grammar and wording of the manuscript.

Changes in the manuscript

Introduction: […] Psychosocial risk factors, including long working hours or and employment uncertainty, are strongly associated with health issues such as burnout, coronary heart diseases, and anxiety […] (lines 59 ff.)

Introduction: […] The findings from this study could help design more favorable and sustainable psychosocial working conditions in organizations that are similar to the one investigated. Designing working conditions based on the learnings from the COVID-19 pandemic can help ensure workability during future challenging times. (lines 126 ff.)

Methods: […] This allowed us to gain detailed insights into the company-level context in which employees and managers work. (lines 145 ff.)

Results: […] Fig 2a visualizes the perceived psychosocial ratings, differentiated per examined working field. (lines 340 f.)

Results: […] Because of established procurement channels and financial resources within the company group, it was possible to carry out large-scale SARS-CoV-2-testing within a short timeframe. (lines 364 f.)

Comment 3

Engagement with Data: You refer to survey findings and telephone interviews but do not provide much detail on the data collection process. Briefly mentioning the survey method (e.g., sample size, how it was administered) could help the reader better understand the research design.

Answer 3

Many thanks for that comment. We can see your point and agree. However, as part of the overall study project (see methods section 'study design') a study protocol, one paper explaining in detail the quantitative methods including survey design and recruiting process as well as one related results paper have already been published. These are cited as '(blinded reference)' throughout the manuscript but have been temporarily removed for the double-blind review process.

To enhance clarity, we now added relevant background information to the present mixed-methods manuscript. For more details, we refer to previously published work. Regarding the sample size, in the overall survey, N=5,554 employees participated. For 322 out of them, longitudinal panel data could be ensured. See section 'Results sample characteristics' for further details, lines 274 ff.. We added an additional sensitivity analysis to show that the outcome variables did not differ substantially between participants who took part three times compared to the overall sample, see S5 Tab in the Supplementary Material. Regarding the qualitative interviews, we considered a sample of nine interview participants sufficient to reach data saturation.

Changes in the manuscript

Methods section 'Quantitative data collection and analysis': […] Quantitative self-reported employee survey data were generated by distributing an online survey three times after the onset of the COVID-19 pandemic in Germany: August 10 to October 25, 2020 (T0), January 21 to 31, 2021 (T1), and October 15 to November 21, 2021 (T2). The questionnaire was designed using the online survey tool Unipark provided by Questback AS [15]. Survey invitations including an access link and QR code were sent to all employees by the company's corporate communications department via email, newsletter, intranet, postcards, and posters, see (blinded reference) for more details. Participation was voluntary and we used convenience sampling. Multiple observations were linked to the same individual using self-generated anonymized codes. Details on recruiting strategies, surveyed items, and response rate at T0 (22%) are described elsewhere (blinded reference). […] (line 193 ff.)

Results section: […] The here presented survey results are based on a subsample of participants (n=322 out of N=5,554) for whom all three survey observations were available. A total of 5,554 employees participated in the online survey at one, two, or all three timepoints. For 322 out of them, longitudinal panel data could be ensured. This analysis focuses on panel data, as we were primarily interested in within-person changes in psychosocial demands. The outcome variables and socio-demographic characteristics did not differ substantially between participants who took part three times compared to the overall sample, see S5 Tab for an additional sensitivity analysis. (lines 275 ff.)

Comment 4

Link Between Concepts: The transition from organizational responses to employee health could be more seamless. Right now, it jumps from one topic to another without much elaboration. It may be helpful to add a sentence or two linking how the organizational responses directly influence employee perceptions of psychosocial demands.

Answer 4

Many thanks for this helpful remark. Your are right. We now hope to have clarified how the COVID-19 pandemic and related infection control measures lead to changes in working conditions and associated psychosocial demands. Furthermore, we aimed to show how organizations can influence the employees' experiences and help buffer negative psychosocial demands throughout the dynamic course of the pandemic.

Changes in the manuscript

Introduction: […] Comprehensive measures were introduced to prevent SARS-CoV-2 infections at work, outbreaks at work spreading to families, a high rate of sick leave, and overwhelmed healthcare systems. Workplaces were adapted: for example re-arrangement to maintain a distance of 1.5m between workstations (technical), remote work where possible (organizational), and the wearing of face masks if distance could not be maintained (personal measures). Thus, daily working conditions changed rapidly as a result of the implemented SARS-CoV-2-infection control measures. Physical distancing on-site, increased remote work, and new cleaning and hygiene protocols led to difficulties in communication flows, blurred boundaries between work and private life, and unclear responsibilities. The dynamic pandemic situation required ongoing re-adaptations in OSH-measures. Those continuous changes in how and under which conditions people work, impacted the work-related psychosocial demands employees experienced during the pandemic. […] (line 91 ff.)

Introduction: […] Organizational responses include, for example, communication strategies, approaches to re-designing on-site work environments, and the adaptation of infection control measures tailored to specific working fields. We investigated how organizations addressed pandemic-related challenges, focusing on working conditions. […] (line 121 ff.)

Comment 5

MATERIAL AND METHODS - You mention using both qualitative and quantitative methods, but this section would benefit from a clearer outline of how each method complements the other. For example, you could describe briefly how the qualitative data (e.g., interviews) and quantitative survey data will be integrated in your analysis.

Answer 5

Thank you very much for this important remark. We revised the methods section and provided more details on how quantitative and qualitative data were integrated. In addition, we highlighted the main advantage of the triangulation for our manuscript.

Changes in the manuscript

Introduction: We investigated how organizations addressed pandemic-related challenges, focusing on working conditions. We aimed to match these findings with how employees and managers perceived psychosocial demands during and retrospectively before the COVID-19 pandemic in Germany. Using the combination of qualitative interviews and a quantitative survey, we aimed to gain insights into organizational pandemic preparedness in work design. The findings from this study could help design more favorable and sustainable psychosocial working conditions in organizations that are similar to the one investigated. Designing working conditions based on the learnings from the COVID-19 pandemic can help ensure workability during future challenging times. (lines 124 ff.)

Methods: In a parallel approach, quantitative (employee survey) and qualitative data (interviews with managers) were gathered and analyzed independently. This allowed us to gain detailed insights into the company-level context in which employees and managers work. Findings from the interviews further helped to address how workers of different working fields experienced support from both the German state (e.g., financial aid in the form of temporary short-time work compensation) and their organization (e.g., adjusted work arrangements) during the pandemic. These pluralistic perspectives enable an evaluation of how the organization’s responses to the challenges of the pandemic affected the employees’ daily work. Data integration was realized during sampling, as both, quantitative and qualitative data, were collected via convenience sampling from the same working fields to ensure comparability. Further, integration was realized during the exploratory analysis and interpretation phase. The triangulation of methods allowed us to investigate consistent patterns in perceived psychosocial demands within working fields as well as between employees and managers. The contextual insights, mainly provided by the qualitative data, allowed a more nuanced understanding of these patterns. (line 143 ff.)

Comment 6

Clarity in Structure and Flow: The first two sentences are somewhat dense and can be broken up for better readability.

“This study is part of a larger explorative modular mixed-methods study project examining the acceptance of occupational SARS-CoV-2-infection control measures (ICM) (blinded).”

This could be more direct: “This study is part of a larger, explorative, modular mixed-methods research project that investigates the acceptance of SARS-CoV-2 infection control measures (ICM) in occupational settings (blinded).”

Clarify "Blinded" Information: You mention "blinded" but do not specify what is blinded. Is it the participant group, the intervention, or something else? It would be clearer if this were explicitly stated.

Answer 6

Thank you. We shortened the sentence describing the study design accordingly. By ‘blinded’ we refer to the the citation of the study protocol, which was published earlier. Due to the double-blind review process, we have removed the reference for now and will add it in case the manuscript gets accepted. The study protocol provides detailed information about the study design and modular mixed-methods approach (see Comment 3 for further explanation).

Changes in the manuscript

Methods section: This study is part of a larger, explorative, modular mixed-methods study project that examines the acceptance of SARS-CoV-2-infection control measures (ICM) in occupational settings (blinded reference). (line 137 ff.)

Comment 7

Provide More Detail on the Mixed-Methods Approach: The description of the mixed-methods approach can be more detailed. For example:

How were the quantitative surveys designed (e.g., Likert scales, multiple-choice questions)?

What was the interview process for the qualitative data? Were there semi-structured interviews, and how were these analyzed (e.g., thematic analysis)?

Providing more insight into these aspects would help the reader understand the depth of your approach.

Answer 7

We hope we now went into more detail in the methods section. We aimed to provide relevant information for a clear understanding when reading this manuscript.

However, as part of the overall study project (see methods section 'study design') a study protocol, one paper explaining in detail the quantitative methods including survey design and recruiting process as well as one related results paper have already been published, see Comment 3 for further explanation. These are cited as "(blinded reference)" throughout the manuscript but have been temporarily removed for the double-blind review process.

Regarding the interview data collection, we already stated that semi-structured interviews were conducted and that data was analyized via qualitative content analysis. We considered a sample of nine interview participants sufficient to reach data saturation. We tried to clarify the methodology used for the interview data analysis.

Changes in the manuscript

Methods section 'Quantitative data collection and analysis': […] The questionnaire was designed using the online survey tool Unipark provided by Questback AS [15]. Survey invitations including an access link and QR code were sent to all employees by the company's corporate communications department via email, newsletter, intranet, postcards, and posters, see (blinded reference) for more details. Participation was voluntary and we used convenience sampling. Multiple observations were linked to the same individual using self-generated anonymized codes. Details on recruiting strategies, surveyed items, and response rate at T0 (22%) are described elsewhere (blinded reference). […] (line 196 ff.)

Methods section 'Quantitative data collection and analysis': […] The survey items were self-developed following the GDA recommendations [7] and Copenhagen Psychosocial Questionnaire (COPSOQ) [16]. Each item was to be rated on a 5-point Likert scale, symmetrically anchored with '3', which indicates a neutral perception. Some items were reversed for score computation, see S1 Tab for more details. […] (line 207 f.)

Methods section 'Qualitative data collection and analysis': […] Semi-structured interviews were conducted from September 28 to October 19, 2020 (t0) and from April 19 to May 6,

2021 (t1) via video call or telephone (24-60 minutes) by author 2 (blinded name). […] Author 2 arranged interview appointments. All interviews began with a brief introduction, information about data protection, and the interview structure. […] The qualitative data were organized using MAXQDA 2020 (VERBI Software, Germany) and analyzed via qualitative content analysis [21] to identify and structure the main topics and sub-topics of the data (S4 Tab). Regarding data saturation, the interviews showed a strong information power [20]. […] (lines 241 ff.)

Comment 8

Sampling Details: You mention that participants come from various working fields but do not specify how they were selected.

“Employees and managers from various working fields...” could benefit from clarification on the sampling method (e.g., purposive sampling, random sampling). Were these employees chosen based on specific criteria?

Additionally, it would be useful to include the sample size for both the employee survey and the interviews with managers.

Participant Inclusion Criteria: The inclusion criteria (e.g., minimum age of 18, B1-level German language proficiency, and informed consent) are clear, but consider adding any exclusion criteria (e.g., those with severe language barriers or any other reasons for exclusion).

Consider rephrasing for clarity: “All participants were at least 18 years old, had a German language proficiency of at least B1, and provided written informed consent.”

Ethical Considerations: The mention of ethical approval is good, but consider providing more detail on the ethical guidelines followed, such as whether any anonymization or confidentiality measures were implemented.

Answer 8

Thank you for your suggestions. We now went more into detail regarding the in- and exclusion criteria, the recruiting process, the sampling method, and sample size. We mainly used convenience sampling. We rephrease the inclusion criteria for a better understanding based on your recommendation.

Please see Comment 3 for further explanation. We will add details to the ethical approval in l. 141, as soon as the double-blind review process is finished. We will then name the ethics committee having provided approval, add the approval number, and state the date of approval.

We added a paragraph in the results section to describe the sample characteristics for the employee survey and interviews.

Changes in the manuscript

Methods section on study design: […] All participants were at least 18 years old, had a German language proficiency of at least B1, and provided written informed consent. Employees were eligible as interview partners if they had been employed for at least six months and were either member of the crisis management team or held an operational management position. No explicit exclusion criteria were defined beyond not meeting the pre-defined inclusion criteria. Due to the limited timeframe between the onset of the pandemic and the first wave of data collection, the survey was administered solely online. As a consequence, there may have been systematic exclusion of individuals without sufficient time

or resources to participate such as internet or email access at work. This was previously discussed in (limited reference). Ethical approval was obtained. The study was conducted in accordance with the Declaration of Helsinki and relevant institutional guidelines. Qualitative data were pseudonymized and quantitative data were anonymized prior to analysis. (l. 193 ff.)

Methods section on quantitative data collection and analysis: The questionnaire was designed using the online survey tool Unipark provided by Questback AS [15]. Survey invitations including an access link and QR code were sent to all employees by the company's corporate communications department via email, newsletter, intranet, postcards, and posters, see (blinded reference) for more details. Participation was voluntary and we used convenience sampling. Multiple observations were linked to the same individual using self-generated anonymized codes. (l. 196 ff.)

Methods section on qualitative data collection and analysis: […] Potential interview partners were suggested by the company's research partners based on a minimum employment time with the company of six months. Interview partners were eligible if they were either members of the crisis management team or holding an operational management position. Author 2 arranged interview appointments. […] (l. 248 ff.)

Results: The here presented survey results are based on a subsample of participants (n=322 out of N=5,554) for whom all three survey observations were available. A total of 5,554 employees participated in the online survey at one, two, or all three timepoints. For 322 out of them, longitudinal panel data could be ensured. This analysis focuses on panel data, as we were primarily interested in within-person changes in psychosocial demands. The outcome variables and socio-demographic characteristics did not differ substantially between participants who took part three times compared to the overall sample, see S5 Tab for an additional sensitivity analysis. See (blinded reference), for a pooled analysis of all available repeated cross-sectional and longitudinal panel data investigating the employees' attitudes toward organizational infection control measures in the workplace. Individual and work-related sample characteristics are shown in Table 1. Eight male and one female managers participated in the interviews. Of these, n=7 interview partners participated in the initial and follow-up interviews. The following perspectives were presented: factory security service, company medical service, works council, technical operation manager, corporate financial management, human resources, and assembly line/ manufacturing. Employees worked at six company sites in three German federal states of one large company group. This allows differentiation between working fields even with small sample sizes and thereby covering possible differences in safety cultures or local pandemic situations. (l. 275 ff.)

Comment 9

Result & Discussion - Clarity and Coherence:

The discussion effectively presents the findings but could benefit from clearer transitions between different sections. For instance, the shift from describing study results to comparing them with other research could be smoother. Consider using transition phrases like "In line with previous findings..." or "Consistent with prior studies, our results suggest..."

Answer 9

You are right. We went through the manuscript and hope to have written transitions between sections in a smoother way.

Changes in the manuscript

Discussion section: […] In comparison to us, Casjens et al. [28] examined psychosocial demands as well as strain. (l. 451 f.)

Discussion section: […] Similar to our findings, it was previously shown in healthcare organizations, that communication was key to making employees feel safe and cared for in their workplaces. (l. 506 f.)

Discussion section: […] Our findings correspond to a study evaluating pilot COVID-19-vaccination campaigns in multiple companies in Germany. (l. 528 f.)

Discussion section: […] This aligns with the findings of Wagner et al. [44], who similarly observed different response patterns when investigating psychosocial demands among nurses compared to physicians. (l. 586 f.)

Comment 9

Depth of Analysis: While the study highlights key psychosocial demands, it would be beneficial to provide a more nuanced interpretation of why no significant changes were observed over time. Were there specific coping mechanisms, employer interventions, or cultural factors that may have contributed to this stability?

Comparisons with Existing Literature: The discussion compares findings with other studies, which strengthens its validity. However, some comparisons (e.g., with anxiety and depression symptom trends) seem somewhat broad. It would be helpful to specify how these studies align with your research focus on work-related psychosocial demands.

Contextualization of Findings: The section on German social security measures is relevant but could be more directly linked to your findings. Did employees who feared job loss despite holding permanent contracts also exhibit higher psychosocial stress? If so, how does this inform policy or workplace interventions?

Answer 9

Thank you very much for this encouraging remark to provide more interpretation even though we have not directly measured these factors. We now come up with new hypothesis evolving from our findings.

And thank you for this important observation to clearly differentiate between psychosocial demands and strain. In our manuscript, we aimed to distinguish between both. As we only assessed psychosocial demands such as work-privacy conflicts, negative work-related stress, and work-related SARS-CoV-2-infection risk, our comparisons were limited to those. In addition, we wanted to highlight other studies' findings linking those demands to strain during the pandemic. We have revised the related paragraph for clarification.

As 99.7% of the participants hold permanent work contracts, it is not possible to compare their psychosocial demands with employees holding fixed-term contracts. However, this is a good point for a nuanced interpretation of the constant psychosocial demands and how work contract characteristics are linked with national interventions.

Changes in the manuscript

Discussion: […] Our results are similar to another panel study among employees not working in the healthcare sector in Germany, where reported psychosocial demands from reduced contact with colleagues and work-privacy conflicts decreased from winter and spring 2021 until winter 2022 [28]. In comparison to us, Casjens et al. [28] examined psychosocial demands as well as strain. They found that psychosocial demands such as work-privacy conflicts, negative work-related stress, and work-related SARS-CoV-2-infection risk were associated with negative strain, here anxiety- and depression-related symptoms. Reported severity of anxiety- and depression-related symptoms increased at the beginning of the COVID-19 pandemic, were highest in winter and spring 2021 but declined until winter 2022 [25]. […] (l. 448 ff.)

Discussion section: […] Our interviews revealed that employees across all working fields initially had existential concerns and feared job loss due to the uncertain global economic situation. Our quantitative data showed that 99.7% of all participants hold permanent work contracts. Thus, we were unable to compare their perceived psychosocial demands with employees holding fixed-term contracts. We can only hypothesize that the job security provided by the permanent work contracts across all working fields in combination with the possibility to work a reduced number of hours with compensation for missing net wage contributed to the stability of perceived work-related psychosocial demands. (l. 471 ff.)

Comment 10

Limitations and Future Research Directions: The discussion does not explicitly address the limitations of the study. Consider adding a paragraph acknowledging potential limitations (e.g., sample size, generalizability, or reliance on self-reported data) and suggesting areas for future research.

Answer 10

Thank you for your comment. We now added additional limitations to our study. In line 591, however, we have already mentioned the limitation regarding self-reported survey data.

Changes in the manuscript

Discussion: […] Evidence suggests that the larger the organization, the more personnel, structured communication approaches, and formalized procedures are available for managing psychosocial risks at work [35, 36]. This limits the generalizability of our findings. The investigated company differs from small or medium-sized companies, where limited financial capacities and lack of experts may restrict organizational resilience. (l. 509 ff.)

Discussion: […] As the German labor system benefitted from strong social security measures that were established already prior to the COVID-19 pandemic, the generalizability of our findings to other countries is limited. In countries other than Germany with weaker social security measures, it seems to be even more important for organizations to design and establish resilient work design to protect employees from severe psychosocial demands during future crises. During the COVID-19 pandemic, e.g., company medical personnel played an important role in the organizational context. This established expertise may also be critical in future public health crises. (l. 532 ff.)

Discussion section ‘strengths and limitations’: […] Regarding limitations, we examined the perceived psychosocial demands among the employees of a large company group characterized by a well-established safety culture and large financial and human resources. The company group made it possible to produce disinfectants and protective equipment in-house at the pandemic’s beginning to reduce supply shortages by equipping suppliers and own employees appropriately. Therefore, our findings may not fully be generalizable to small or medium-sized organizations with more constrained financial, technical, and human resources, as already discussed in further detail in (blinded reference). Another limitation is the reliance on a small number of recommended dimensions for implementing a comprehensive psychosocial risk assessment at work by the GDA [7]. Results are limited in the transferability to other countries as the GDA-scheme refers to the German legislation and context. Cronbach’s alpha (α=0.5) of the aspect of work content shows a low homogeneity of the three test items. Here, different response patterns of employees of different working fields should be considered. This aligns with the findings of Wagner et al. [44], who similarly observed different response patterns when investigating psychosocial demands among nurses compared to physicians. The low Cronbach’s alpha highlights the importance of interpreting results working field-specifically, considering the work context of each. In terms of methodological

limitations, the quantitative data relied on a self-reported survey; hence, common method biases may exist, including, e.g., possible sample bias. (l. 573 ff.)

Comment 11

Practical Implications:

The findings suggest key organizational strategies (e.g., crisis management teams, participatory approaches). It would be useful to elaborate on how these strategies can be applied beyond the COVID-19 context to improve workplace well-being in future crises.

Answer 11

We appreciate this comment and now elaborated on the advantages of having developed and implemented pandemic preparedness plans for future crises and added an additional paragraph.

Changes in the manuscript

Practical implications: […] During the COVID-19 pandemic, e.g., company medical personnel played an important role in the organizational context. This established expertise may also be critical in future public health crises. (l. 537 ff.)

Future work: […] From our point of view, the here presented findings can be transferred to other current and future global challenges requiring a high level of employee-engagement in organizations. Global challenges such as climate change, economic downturns, or topics related to demographic change have in common that they disrupt established routines and require decision-making under uncertain and dynamic conditions. In our opinion, pandemic preparedness plans not only strengthen organizational resilience during pandemic-like situations. Established structures and strategies further allow faster and solution-driven reactions during other future crises. On April 17, 2025, the WHO member states finalized a draft regarding pandemic preparedness to strengthen global collaboration such as ensuring stable supply chains of medical goods and to highlight the importance of developing pandemic preparedness plans on a national level [45]. (line 599 ff.)

Future work: […] Topics for future research on the topic of demographic changes could deal for example with how managers address digital competency gaps among older workers or how to support those workers' lifelong learning in rapidly changing work environments. (lines 618 ff.)

Comment 13

Clarification of Multi-Level Approach: The section discusses macro-, meso-, and micro-level responses, but the distinction between these levels is sometimes unclear. Consider adding a brief introductory sentence explaining how each level interacts and contributes to overall organizational resilience.

For example: "Our findings highlight the importance of a multi-level response to crisis management, where macro-level policies provide the framework, meso-level decisions implement changes, and micro-level adaptations ensure individualized solutions."

Answer 13

Thank you for your comment. We added a brief introductory sentence following your recommendation.

Changes in the manuscript

Discussion section ‘practical implications’: To better understand how working conditions can ensure workability during challenging times, we aimed to derive practical implications for organizational pandemic preparedness from our findings (meso-level (company level)). As interactions between players exist [31], we additionally draw learnings on the macro- (societal level) and micro-level (individual level). While macro-level policies usually provide a certain framework, changes are implemented on the meso-level to ensure health-related preventive behaviors on the micro-level. (lines 486 ff.)

Comment 14

Stronger Link to Broader Workplace Implications: The discussion primarily focuses on the specific organization studied. To make the findings more generalizable, explicitly address how these lessons can apply to organizations of varying sizes, industries, and resource levels.

Comparing to International Contexts: The practical implications are framed within the German labor system, which benefits from strong social security measures. Briefly acknowledge how organizations in countries with weaker labor protections might face different challenges and require alternative strategies.

Answer 14

Thank you for your comment. You are right regarding the limited generalizability of our findings to small companies or companies located in other countries than Germany. We hope to now have elaborated on the generalizability of our findings.

We already discussed the limitation of generalizability to small or medium-sized companies in line 461 ff. and hope to have now clarified the suggestion of cooperation between smaller organizations or within employer associations to compensate for limited financial capacities and human resources. This was already stated in a pandemic plan for non-healthcare organizations provided for example by the German Social Accident Insurance in cooperation with the Federal Office for Civil Protection [37].

Changes in the manuscript

Discussion: […] As the German labor system benefitted from strong social security measures that were established already prior to the COVID-19 pandemic, the generalizability of our findings to other countries is limited. In countries other than Germany with weaker social security measures, it seems to be even more important for organizations to design and establish resilient work design to protect employees from severe psychosocial demands during future crises. During the COVID-19 pandemic, e.g., company medical personnel played an important role in the organizational context. This established expertise may also be critical in future public health crises. […] (l. 532 ff.)

Practical implications: […] Evidence suggests that the larger the organization, the more personnel, structured communication approaches, and formalized procedures are available for managing psychosocial risks at work [35, 36]. This limits the generalizability of our findings. The investigated company differs from small or medium-sized companies, where limited financial capacities and lack of experts may restrict organizational resilience [37]. Cooperation between smaller organizations or within employer associations could compensate for this. This suggestion was already stated in a pandemic plan for non-healthcare organizations provided for example by the German Social Accident Insurance in cooperation with the Federal Office for Civil Protection [38]. (lines 509 ff.)

Comment 15

Strengths and Limitations - Addressing Possible Biases More Explicitly:

The discussion acknowledges recall bias and negativity bias but could be more explicit in explaining how these biases might have influenced results. For example, were there any trends in the qualitative data that suggest participants' perceptions changed over time?

Also, the discussion of Cronbach's alpha (α=0.5) for the work content aspect suggests low reliability. How does this impact the interpretation of results?

Limitations on Generalizability: The study is based on a single company with pre-existing structural resources. This should be explicitly mentioned as a limitation in terms of external validity, particularly for small and medium-sized enterprises (SMEs).

Consider adding: "The findings may not fully generalize to SMEs or organizations in different sectors where financial and human resources are more constrained."

Answer 15

Thank you for the detailed comment. We hope that we have now provided more clarity on how recall bias and negativity bias could have influenced results. Thank you very much for pointing out that the qualitative data may also suggest recall and negativity bias.

To clarify that the study is based on a single company located in Germany, we additionally adapted to title.

Changes in the manuscript

Discussion section: Both recall and negativity bias may have contributed to an overestimation of unfavorably perceived working conditions during the pandemic compared to before, when rating the 'before' timepoint retrospectively. Evidence of this bias was apparent not only in the quantitative data but also in the qualitative findings. For example, one interviewee reflecting on the last year (IP8a), emphasized intense negative emotions related to fear about health and economic uncertainty. This illustrates how strong intense negative emotional experiences might have shaped the retrospective evaluations. However, our results did not show any significant changes over time. In addition, the retrospective evaluations gathered in 2020 and 2021 presented here, seem to show a similar snapshot of the company's employees' perceived well-being at work compared to before the pandemic. According to the internal results of a worldwide employee survey at the collaborating company with a response rate of 86% (n=270.000) in 2017, the majority of participants were satisfied with their working conditions and 82% would even recommend their employer to family and friends. (line 564 ff.)

Discussion section 'strengths and limitation': Cronbach's alpha (α=0.5) of the aspect of work content shows a low homogeneity of the three test items. Here, different response patterns of employees of different working fields should be considered. This aligns with the findings of

Wagner et al. [44], who similarly observed different response patterns when investigating psychosocial demands among nurses compared to physicians. The low Cronbach's alpha highlights the importance of interpreting results working field-specifically, considering the work context of each. (l. 591 ff.)

Discussion section 'strengths and limitation': Regarding limitations, we examined the perceived psychosocial demands among the employees of a large company group characterized by a well-established safety culture and large financial and human resources. The company group made it possible to produce disinfectants and protective equipment in-house at the pandemic's beginning to reduce supply shortages by equipping suppliers and own employees appropriately. Therefore, our findings may not fully be generalizable to small or medium-sized organizations with more constrained financial, technical, and human resources, as already discussed in further detail in (blinded reference). (l. 580 ff.)

Title: Pandemic preparedness in shaping psychosocial working conditions – insights for occupational safety and health from a longitudinal mixed-methods study during the COVID-19 pandemic at six company sites of one organization in Germany (l. 1 ff.)

Comment 16

Future Work - More Specific Recommendations for Future Studies: The section discusses potential extensions of this research, particularly in relation to demographic change. However, it would be helpful to outline specific research questions or methodologies that future studies could use.

Expanding the Scope Beyond Pandemics: The discussion touches on other global challenges (e.g., demographic changes). It would be helpful to explicitly state how the findings might inform crisis management for challenges like climate change, economic downturns, or geopolitical instability.

Answer 16

We appreciate your encouraging remark to elaborate on future challenges where findings could help to make organizations more resilient. We additionally added a reference for citing the very new draft regarding pandemic preparedness to strengthen global collaboration by the WHO member states finalized mid April, 2025.

Changes in the manuscript

Future work section: Demographic change and its implications for the world of work are already very prominent in Germany. Previous study findings for a representative German population sample revealed that the majority of study participants, born in 1959 or 1964, reported that they were not willing to work until retirement age [46]. The findings additionally showed that when employees perceived improved working conditions and favorable safety culture, they were more likely to delay retirement. Examined aspects of work design included, e.g., feeling appreciated for their work or a perceived personal responsibility in terms of work-time organization. Topics for future research on the topic of demographic changes could deal for example with how managers address digital competency gaps among older workers or how to support those workers' lifelong learning in rapidly changing work environments. (lines 615 ff.)

Future work section: Global challenges such as climate change, economic downturns, or topics related to demographic change have in common that they disrupt established routines and require decision-making under uncertain and dynamic conditions. In our opinion, pandemic preparedness plans not only strengthen organizational resilience during pandemic-like situations. Established structures and strategies further allow faster and solution-driven reactions during other future crises. On April 17, 2025, the WHO member states finalized a draft regarding pandemic preparedness to strengthen global collaboration such as ensuring stable supply chains of medical goods and to highlight the importance of developing pandemic preparedness plans on a national level [45]. (l. 605 ff.)

Comment 17

Conclusions - Clearer Takeaways for Different Stakeholders:

The conclusion emphasizes organizational preparedness but could be more actionable by addressing specific stakeholders. Consider framing the key takeaways separately for policymakers, managers, and employees.

Answer 17

Thank you for your encouraging comment to address specific stakeholders in a more active manner. We have tried to phrase the key takeaways more concretely.

Changes in the manuscript

Future work section: Derived practical implications are particularly relevant for policymakers, managers, and company's medical service personnel. On an organizational level, for managers, derived practical implications highlight the importance of transparent communication, trust-based leadership styles, and participatory approaches in change processes. For company's medical service personnel, it again became clear that they played a key role in creating a certain culture of trust by contributing to decisions of the crisis management team and managing internal testing and vaccination campaigns as part of the national vaccination strategy. The hereby established culture of trust and expertise may also be critical in future public health crises. (line 634 ff.)
