# Supplementary material for: Pandemic preparedness in shaping psychosocial working conditions – insights for occupational safety and health from a longitudinal mixed-methods study during the COVID-19 pandemic at six company sites of one organization in Germany
Source: PLoS One. 2025 Aug 11;20(8):e0328410. doi: 10.1371/journal.pone.0328410 (PMC12338823; doi:10.1371/journal.pone.0328410)
Supplement: Attachment — Submitted filename: Response_to_Reviewers_auresp_3.pdf [file pone.0328410.s010.pdf]

Manuscript ID: PONE-D-25-00343

Title: Pandemic preparedness in shaping psychosocial working conditions – insights for occupational safety and health from a longitudinal mixed-methods study during the COVID-19 pandemic

Dear Prof. Dr. Damiano Girardi,

thank you very much for having taken the time and effort to carefully read through our manuscript and the previous reviewers' comments. Thank you for your positive and encouraging feedback on our revised manuscript and responses to the reviewers' comments. We are pleased to hear that the revisions have improved the clarity and quality of the work and that most concerns have been satisfactorily addressed. We appreciate your suggestions on the remaining minor issues.

Please find below our answers to the remaining points on our manuscript now titled '*Pandemic preparedness in shaping psychosocial working conditions – insights for occupational safety and health from a longitudinal mixed-methods study during the COVID-19 pandemic at six company sites of one organization in Germany'.*

In addition, we uploaded a marked-up copy of our manuscript to highlight the changes made, labeled 'Revised Manuscript with Track Changes' as well as an unmarked version of the revised manuscript labeled 'Manuscript'.

Thank you for having taken over this review process. We look forward to hearing from you.

Kind regards on behalf of all authors,

Jana Soeder (Corresponding author)

Research Associate and PhD Candidate Experimental Medicine

Institute of Occupational and Social Medicine and Health Services Research

University Hospital Tübingen

Wilhelmstrasse 27

72074 Tuebingen, Germany

Comment 1

p.2, line 21, "This longitudinal mixed-methods study explores how organizations dealt with COVID-19 pandemic-related challenges". The investigation involved workers employed within a single company group, which may limit the generalizability of the findings.

Answer 1

Many thanks for your recommendation. We have revised the sentence describing the objective of our study to avoid creating the false impression that the results can be generalized without limitation.

Changes in the manuscript

Abstract: This longitudinal mixed-methods study explores how one company group dealt with COVID-19 pandemic-related challenges and how employees and managers perceived work-related psychosocial demands in Germany. […] (lines 21-23)

Comment 2

p.4, line 74, "depending on the individual’s preconditions". Why do the authors mean by individual preconditions? Personality traits, socio-demographic characteristics, ... An example should clarify this point.

Answer 2

Many thanks for that comment. We can see your point and agree. We now added three examples to clarify that point.

Changes in the manuscript

Introduction: Psychosocial demands can – depending on the individual’s preconditions such as socio-demographic characteristics, experiences, and current mental state – lead to either favorable (e.g., activating state) or unfavorable strain (e.g., fatigue or problems concentrating) and thus impact the individual’s health [7]. (lines 74-78)

Comment 3

p.7, line 136. This section appears somewhat heterogeneous, as it presents various types of information. In my opinion, the description of the study aims (e.g., 'findings from the interviews further helped to address how workers from different occupational fields experienced...'), the methodology (i.e., the mixed-method approach), and potentially the study's contribution to the field, might be better organized under a dedicated section—for example, titled "The Current Study".

Answer 3

Thank you for that comment. You are right, the section had become difficult to read. We have now restructured it and hope that the different types of information are now presented in a clearer and more understandable way.

Changes in the manuscript

Material and methods:

Study design

This study is part of a larger, explorative, modular study project that examines the acceptance of SARS-CoV-2-infection control measures in occupational settings (blinded reference). Ethical approval was obtained. The study project was conducted in accordance with the Declaration of Helsinki and relevant institutional guidelines.

For the current study, we applied a mixed-methods approach, commonly used in organizational health services research to consider the complexity of organizations [12, 13]. Using the combination of qualitative interviews and a quantitative survey, we aimed to gain insights into organizational pandemic preparedness in work design. Quantitative survey data were collected at multiple time points to monitor changes in how the employees' and managers' perceived psychosocial demands at work. Findings from the interviews further helped to address how workers of different working fields experienced support from both the German state (e.g., financial aid in the form of temporary short-time work compensation) and their organization (e.g., adjusted work arrangements) during the pandemic. These pluralistic perspectives enable an evaluation of how the organization's responses to the challenges of the pandemic affected the employees' daily work.

The findings from this study could help design more favorable and sustainable psychosocial working conditions in organizations that are similar to the one investigated. Designing working conditions based on the learnings from the COVID-19 pandemic can help ensure workability during future challenging times.

Mixed-methods approach

For contextualization, we performed a comprehensive document analysis of prevailing national and federal laws and OSH-regulations for infection control in Germany. In a parallel approach, quantitative (employee survey) and qualitative data (interviews with managers)

were gathered and analyzed independently. This allowed us to gain detailed insights into the company-level context in which employees and managers work. Data integration was realized during sampling, as both, quantitative and qualitative data, were collected via convenience sampling from the same working fields to ensure comparability. Further, integration was realized during the exploratory analysis and interpretation phase. The triangulation of methods allowed us to investigate consistent patterns in perceived psychosocial demands within working fields as well as between employees and managers. The contextual insights, mainly provided by the qualitative data, allowed a more nuanced understanding of these patterns. We report following the GRAMMS-guidelines [14].

Study participants

The longitudinal data comes from employees and managers from various working fields from a worldwide leading global supplier of technology and services. The company group can be characterized as financially well-established and having a lot of human resources (blinded reference). For example, regarding the work environment, digitalized solutions and proven communication channels were already used before the COVID-19 pandemic. All employees and managers included were employed at six German company sites in the federal states of Bavaria, Baden-Wurttemberg, and Lower Saxony. Employees for example worked at the assembly line/production, in the office, company medical service, or factory security service of the same company group. We aim to differentiate between those working fields as they show large differences in how working conditions were adapted to ensure as-safe-as-possible workplaces. While office workers were mainly affected by remote work and online meetings where possible, assembly line/manufacturing workers experienced redesigned working conditions to allow for keeping a minimum of 1.5m distance between work stations, frequent cleaning of work equipment, and decoupled break times. Company medical service personnel in comparison were responsible for implementing SARS-CoV-2-infection control measures as well as ensuring testing and vaccination possibilities. All participants were at least 18 years old, had a German language proficiency of at least B1, and provided written informed consent.

Employees were eligible as interview partners if they had been employed for at least six months and were either member of the crisis management team or held an operational management position. No explicit exclusion criteria were defined beyond not meeting the pre-defined inclusion criteria. Due to the limited timeframe between the onset of the pandemic and the first wave of data collection, the employee survey was administered solely online. As a consequence, there may have been systematic exclusion of individuals without sufficient time or resources to participate such as internet or email access at work. This was previously discussed in (limited reference). Qualitative data were pseudonymized and quantitative data were anonymized prior to analysis. (lines 119-202)

Comment 4

p.21. The authors should clearly acknowledge that, in some cases, the number of participants within specific occupational fields was too small to support statistical inference or allow for generalization of the results.

Answer 4

We added a statement to the 'Limitations' section to acknowledge that the small sample size limits the generalizability of the results.

Changes in the manuscript

Discussion - limitations: […] Office and assembly line work areas generally represent the largest groups of employees within a company, whereas groups such as the company medical service are naturally smaller. However, the sample size in some work areas was too small to draw statistical conclusions or generalize the results. (lines 598-602)
